# The association of six non‐synonymous variants in three DNA repair genes with hepatocellular carcinoma risk: a meta‐analysis

**DOI:** 10.1111/jcmm.12896

**Published:** 2016-06-16

**Authors:** Yan‐Hui Shi, Bin Wang, Bai‐Ping Xu, Dan‐Na Jiang, Dong‐Mei Zhao, Man‐Ru Ji, Li Zhou, Xue Li, Chang‐Zhu Lu

**Affiliations:** ^1^ Department of GastroenterologyThe First Hospital of Qiqihar CityQiqiharHeilongjiangChina; ^2^ Department of PhysiologyQiqihar Medical UniversityQiqiharHeilongjiangChina; ^3^ Intervention Therapy DepartmentThe First Hospital of Qiqihar CityQiqiharHeilongjiangChina; ^4^ Central LaboratoryQiqihar Medical UniversityQiqiharHeilongjiangChina

**Keywords:** hepatocellular carcinoma, DNA repair gene, non‐synonymous variant, risk estimate, meta‐analysis

## Abstract

Hepatocellular carcinoma is a complex polygenic disease. Despite the huge advances in genetic epidemiology, it still remains a challenge to unveil the genetic architecture of hepatocellular carcinoma. We, therefore, decided to meta‐analytically assess the association of six non‐synonymous coding variants from *XRCC1*,*XRCC3* and *XPD* genes with hepatocellular carcinoma risk by pooling the results of 20 English articles. This meta‐analysis was conducted according to the PRISMA statement, and data collection was independently completed in duplicate. In overall analyses, the minor alleles of four variants, Arg280His (odds ratio, 95% confidence interval, *P*: 1.37, 1.13–1.66, 0.001), Thr241Met (1.93, 1.17–3.20, 0.011), Asp312Asn (1.22, 1.08–1.38, 0.001) and Lys751Gln (1.42, 1.02–1.97, 0.038), were associated with the significant risk for hepatocellular carcinoma. There were low probabilities of publication bias for all variants. Subgroup analyses revealed significant association of *XRCC1* gene Arg399Gln with hepatocellular carcinoma in Chinese especially from south China (odds ratio, 95% confidence interval, *P*: 1.57, 1.16–2.14, 0.004), in larger studies (1.48, 1.11–1.98, 0.007) and in studies with population‐based controls (1.33, 1.06–1.68, 0.016). Taken together, our findings demonstrated that *XPD* gene Asp312Asn and *XRCC1* gene Arg399Gln might be candidate susceptibility loci for hepatocellular carcinoma. Considering the ubiquity of genetic heterogeneity, further validation in a broad range of ethnic populations is warranted.

## Introduction

Liver cancer is rapidly escalating to epidemic proportions worldwide, and it constitutes a major burden on individual and public health. Liver cancer is much more common in men than in women, and it is a common malignancy in Asia and Africa 1. The most common type of liver cancer is hepatocellular carcinoma, which accounts for more than 70% of cases 2. Despite the huge advances in genetic epidemiology, to unveil the genetic architecture of hepatocellular carcinoma still remains a challenging task, as apparent inconsistency from many individual underpowered studies has clouded the true effect of culprit genotype 3, 4. A practical and popular way is to pool the results of these individual studies to generate a more reliable estimate, just as a meta‐analysis does.

DNA damage is widely recognized as a major primary cause of cancer, and without correct repair, such damage can give rise to genetic instability and eventually carcinogenesis 5, 6. Individuals differ greatly in their ability to repair DNA damage, and it is of clinical importance to interrogate DNA damage repair genes to account for this interindividual difference. The task of moving from the selection of candidate DNA repair genes to the enumeration of causal variants poses a challenge. It represents a useful shortcut to interrogate a non‐synonymous coding variant with strong biological credentials in a compelling gene. To generate convincing information, we in this study decided to employ a meta‐analytical method to assess the association of six widely evaluated non‐synonymous coding variants from three DNA repair genes, that is, *XRCC1* (gene ID: 7515), *XRCC3* (gene ID: 7517) and *XPD* (gene ID: 2068), in susceptibility to hepatocellular carcinoma, by pooling the summarized data from 20 English articles from medical literature.

## Methods

### Search strategies

Identification of relevant articles investigating the association of *XRCC1*,* XRCC3* or *XPD* genetic variant(s) with the risk for hepatocellular carcinoma was carried out through searching PubMed and Embase using the predefined key words: (‘hepatocellular’ or ‘liver cancer’ in Title) and (‘XRCC*’ or ‘XPD’ or ‘DNA damage repair’ or ‘DNA repair’ in Title/Abstract) and (‘genotype’ or ‘allele’ or ‘polymorphism’ or ‘variant’ or ‘mutation’ in Title/Abstract). The latest search checkpoint was on October 19, 2015. Each source of relevant articles was integrated into the EndNote X5 software (Thomson Reuters EndNote, Times Square, NY, USA) for reference management. In addition, the bibliographies of some major narrative reviews, original and meta‐analytical articles were also abstracted to make sure there was no missing hit. The conduct of this meta‐analysis was in agreement with the guidelines formulated in the Preferred Reporting Items for Systematic Reviews and Meta‐analyses (PRISMA) statement (Table S1) 7.

### Inclusion/exclusion criteria

Articles were included if one or more of the six variants (*XRCC1* gene: Arg194Trp, Arg280His, Arg399Gln; *XRCC3* gene: Thr241Met; *XPD* gene: Asp312Asn, Lys751Gln) were examined in association with hepatocellular carcinoma risk, and the count of each genotype was provided in both hepatocellular carcinoma patients and controls. In case of unavailable genotype counts, the article was includable if odds ratio (OR) and its 95% confidence interval (95% CI) were provided. Articles were excluded if they were conference abstracts or posters due to insufficient information or if they were editorials, narrative reviews, meta‐analyses or lacking of control groups.

### Data collection

Using a uniform Excel form, two authors (Yan‐Hui Shi and Bin Wang) independently collected and cross‐checked all necessary data from each qualified article, with any disagreement solved by a discussion. All necessary data included surname of the first author, year of publication, country where study samples collected and their acquisition time, sources of patients and controls, genotyping method, matched status, sample sizes, the genotype counts of six examined variants between the two groups and some baseline characteristics, including age, gender, smoking, drinking, hepatitis B and C virus infection, and family history of cancer.

### Statistical analysis

Given the small number of mutant homozygous genotypes, the association of six examined variants with hepatocellular carcinoma risk was assessed under allelic and dominant models only. The random‐effects model using the DerSimonian and Laird method was employed to compute weighted OR and its 95% CI. The inconsistency index (*I* ^2^) was used to quantify the magnitude of heterogeneity across studies, and the *I* ^2^, which takes values from 0% to 100%, represents the percent of the observed variability that results from heterogeneity rather than chance. Practically, the *I* ^2^ of greater than 50% was indicative of significant heterogeneity.

To account for the potential sources of clinical heterogeneity, some stratified analyses were undertaken according to country, source of controls, sample size, genotyping method and matched status, respectively. Only subgroups with three or more independent studies were presented. In addition, a meta‐regression analysis was used to detect other sources from some continuous confounders, including age, gender, smoking, drinking, hepatitis B and C infection, and family history of cancer. In addition, sensitivity analysis was performed by removing an individual study each time to check whether it can bias the overall estimate.

The probability of publication bias was assessed by the trim‐and‐fill method, which was used to assess the potential effect of missing studies with negative findings that may have had on the observed estimates.

The above analyses were completed by Stata software version 12.0 for Windows (StataCorp, College Station, TX, USA).

## Results

Using the predefined key words, we have identified a total of 78 potential relevant articles from PubMed and Embase databases. Through a rigorous process of selection, 20 English articles involving 20 independent studies (6449 hepatocellular carcinoma patients and 8263 controls) were qualified for the final analysis 8, 9, 10, 11, 12, 13, 14, 15, 16, 17, 18, 19, 20, 21, 22, 23, 24, 25, 26, 27, and the selection process is presented in Fig. S1. The majority of qualified studies were from China (*n* = 14) and the Indo‐Pakistani region (*n* = 4). There were, respectively, 6, 4, 13, 6, 4 and 6 studies for variants Arg194Trp, Arg280His, Arg399Gln, Thr241Met, Asp312Asn and Lys751Gln. The baseline characteristics of these 20 studies are presented in Table 1.

**Table 1:** The baseline characteristics of 20 qualified studies in this meta‐analysis

Author (year)	Country	Duration	Sources		Genotyping	Matched status	Sample size		Age (years)		Male (%)		HBV (%)		HCV (%)		Smoking (%)		Drinking (%)		Family history of cancer (%)	
			Patients	Controls			Cases	Cont's	Cases	Cont's	Cases	Cont's	Cases	Cont's	Cases	Cont's	Cases	Cont's	Cases	Cont's	Cases	Cont's
Yang *et al*. (2015)	China‐Shandong	January 2010 to August 2014	1 hospital	Hospital	RFLP	Age, sex, residence	118	120	NR	NR	72.0	60.8	NR	NR	NR	NR	33.9	15.8	41.5	14.2	NR	0.0
Yao *et al*. (2014)	China‐Guangxi	January 2004 to December 2012	2 hospitals	Health check‐up centre	TaqMan	Age, sex, ethnicity, HBV, HCV	1486	1996	NR	NR	75.4	76.0	72.9	70.5	18.6	17.8	NR	NR	NR	NR	NR	NR
Wu *et al*. (2014)	China‐Chongqing	May 2006 to October 2008	2 hospitals	Health check‐up centre	Array	Age	218	277	52.2	53.7	65.6	57.4	52.3	7.2	7.4	1.1	34.9	27.8	46.3	36.8	7.8	0.7
Luo *et al*. (2014)	China‐Chongqing	January 2010 to December 2012	1 hospital	Hospital	TaqMan	Age, sex	300	300	55.3	54.1	82.0	81.3	25.7	NR	NR	NR	41.7	39.3	34.7	33.7	5.7	0.0
Mohana *et al*. (2013)	India	NR	Multiple hospitals	Hospital	RFLP	Age, sex, race	93	93	NR	NR	NR	73.1	73.1	NR	NR	NR	NR	NR	NR	NR	NR	0.0
Gulnaz *et al*. (2013)	Pakistan	2007–2009	1 hospital	Hospital	RFLP	NR	50	74	NR	NR	68.0	68.0	22.0	0.0	54.0	0.0	28.0	22.0	4.0	3.0	NR	NR
Bose *et al*. (2013)	India	NR	1 hospital	Hospital	RFLP	Age, sex	55	210	54.0	NR	83.6	NR	NR	NR	NR	NR	NR	NR	0.0	NR	NR	NR
Zeng *et al*. (2012)	China‐Guangxi	August 2007 to November 2008	2 hospitals	Hospital	TaqMan	Age, sex, ethnicity	497	500	NR	NR	78.7	74.2	76.7	11.6	NR	NR	32.8	9.6	39.6	10.0	9.5	0.6
Yuan *et al*. (2012a)	China‐Chongqing	January 2009 to December 2011	1 hospital	Hospital	RFLP	Age, birthplace	350	400	52.1	51.1	76.3	75.0	80.1	79.3	NR	NR	67.8	67.1	78.0	77.5	14.6	13.2
Yuan *et al*. (2012b)	China‐Chongqing	January 2009 to December 2010	1 hospital	Hospital	RFLP	Age, sex	252	250	51.6	52.1	75.8	74.0	80.6	77.6	NR	NR	66.7	67.6	75.4	76.0	14.7	14.0
Han *et al*. (2012)	China‐Shandong	May 2008 to May 2010	1 hospital	Health check‐up centre	CTPP	Age	150	158	51.3	50.8	63.3	62.0	56.7	46.8	11.3	8.2	38.7	31.7	59.3	39.2	NR	0.0
Guo *et al*. (2012)	China‐Liaoning	January 2008 to December 2011	1 hospital	Health check‐up centre	CTPP	Age, sex	410	410	51.5	51.4	65.6	65.6	36.5	8.6	5.1	0.9	36.5	22.8	41.3	31.9	10.7	1.8
Pan *et al*. (2011)	China‐Shandong	May 2008 to May 2010	1 hospital	Health check‐up centre	CTPP	Age	202	236	50.5	50.2	67.3	64.0	52.5	48.7	11.9	9.3	44.1	30.1	62.9	33.1	NR	0.0
Long *et al*. (2009)	China‐Guangxi	January 2006 to August 2008	2 hospitals	Health check‐up centre	RFLP	Age, sex, ethnicity, HBV, HCV	618	712	49.3	49.2	72.5	75.8	72.8	71.3	18.4	18.0	NR	NR	NR	NR	NR	NR
Kiran *et al*. (2009)	India	NR	1 hospital	Relatives or attendants	RFLP	NR	53	142	59.3	32.5	90.5	72.5	NR	0.0	NR	0.0	NR	NR	NR	NR	NR	NR
Long *et al*. (2008)	China‐Guangxi	September 2004 to August 2006	2 hospitals	Health check‐up centre	RFLP	Age, sex, ethnicity, HBV, HCV	491	862	NR	NR	73.7	74.4	73.3	74.8	18.1	15.2	NR	NR	NR	NR	NR	NR
Borentain *et al*. (2007)	France	NR	1 hospital	Hospital	Sequencing	NR	56	77	54.4	48.0	87.5	67.5	31.0	5.0	44.0	27.0	NR	NR	37.0	38.0	NR	NR
Long *et al*. (2006)	China‐Guangxi	January 2004 to May 2005	1 hospital	Health check‐up centre	RFLP	Age, sex, ethnicity, HBV	257	649	NR	NR	80.9	75.5	83.7	80.0	19.1	14.3	NR	NR	NR	NR	NR	NR
Kirk *et al*. (2005)	France	NR	Multiple hospitals	Hospital	RFLP	Age, sex	216	408	48.1	44.8	80.1	71.6	61.1	15.9	18.9	2.9	NR	NR	NR	NR	NR	NR
Chen *et al*. (2005)	China‐Taiwan	January 1997 to December 2001	3 hospitals	A cohort of HBsAg carriers	Array	Age, sex	577	389	52.3	53.0	86.0	86.0	100.0	100.0	NR	NR	NR	NR	NR	NR	NR	NR

RFLP, restriction fragment length polymorphism; NR, not reported; HBV, hepatitis B virus; HCV, hepatitis C virus; Cont's, controls.

Under allelic model, the association of minor alleles of six examined variants with hepatocellular carcinoma risk was statistically significant for Arg280His (OR, 95% CI, *P*: 1.37, 1.13–1.66, 0.001), Thr241Met (1.93, 1.17–3.20, 0.011), Asp312Asn (1.22, 1.08–1.38, 0.001) and Lys751Gln (1.42, 1.02–1.97, 0.038) only, and no indication of heterogeneity was observed for Thr241Met (*I* ^2^ = 16.5%) and Asp312Asn (*I* ^2^ = 0.0%) (Table 2). Similarly, under dominant model, the magnitude of association was strengthened for all variants, and four variants mentioned above along with Arg194Trp exhibited significant association, and there was no heterogeneity for Arg194Trp (*I* ^2^ = 0.0%) and Asp312Asn (*I* ^2^ = 0.0%) only. However, after the Bonferroni correction for multiple tests (*P* < 0.05/6), only Asp312Asn persisted significant under both genetic models (Table 2). In addition, our sensitivity analyses of six examined variants revealed that no individual studies were observed to influence the pooled estimates significantly (data not shown).

**Table 2:** Risk estimates of six examined variants for hepatocellular carcinoma under both allelic and dominant models

Variants	Number of studies	OR	95% CI	*P*	*I* ^2^ (%)
Allelic model					
Arg194Trp	6	1.11	0.96–1.29	0.170	23.7
Arg280His	4	1.37	1.13–1.66	0.001	16.5
Arg399Gln	13	1.19	0.97–1.46	0.093	85.4
Thr241Met	6	1.93	1.17–3.20	0.011	96.1
Asp312Asn	4	1.22	1.08–1.38	0.001	0.0
Lys751Gln	6	1.42	1.02–1.97	0.038	92.7
Dominant model					
Arg194Trp	6	1.23	1.04–1.45	0.016	0.0
Arg280His	4	1.53	1.11–2.10	0.010	52.8
Arg399Gln	13	1.24	0.97–1.58	0.082	81.9
Thr241Met	6	1.84	1.02–3.33	0.043	95.7
Asp312Asn	4	1.24	1.07–1.44	0.005	0.0
Lys751Gln	6	1.49	1.02–2.18	0.042	91.2

OR, odds ratio; 95% CI, 95% confidence interval.

**Figure 1:**
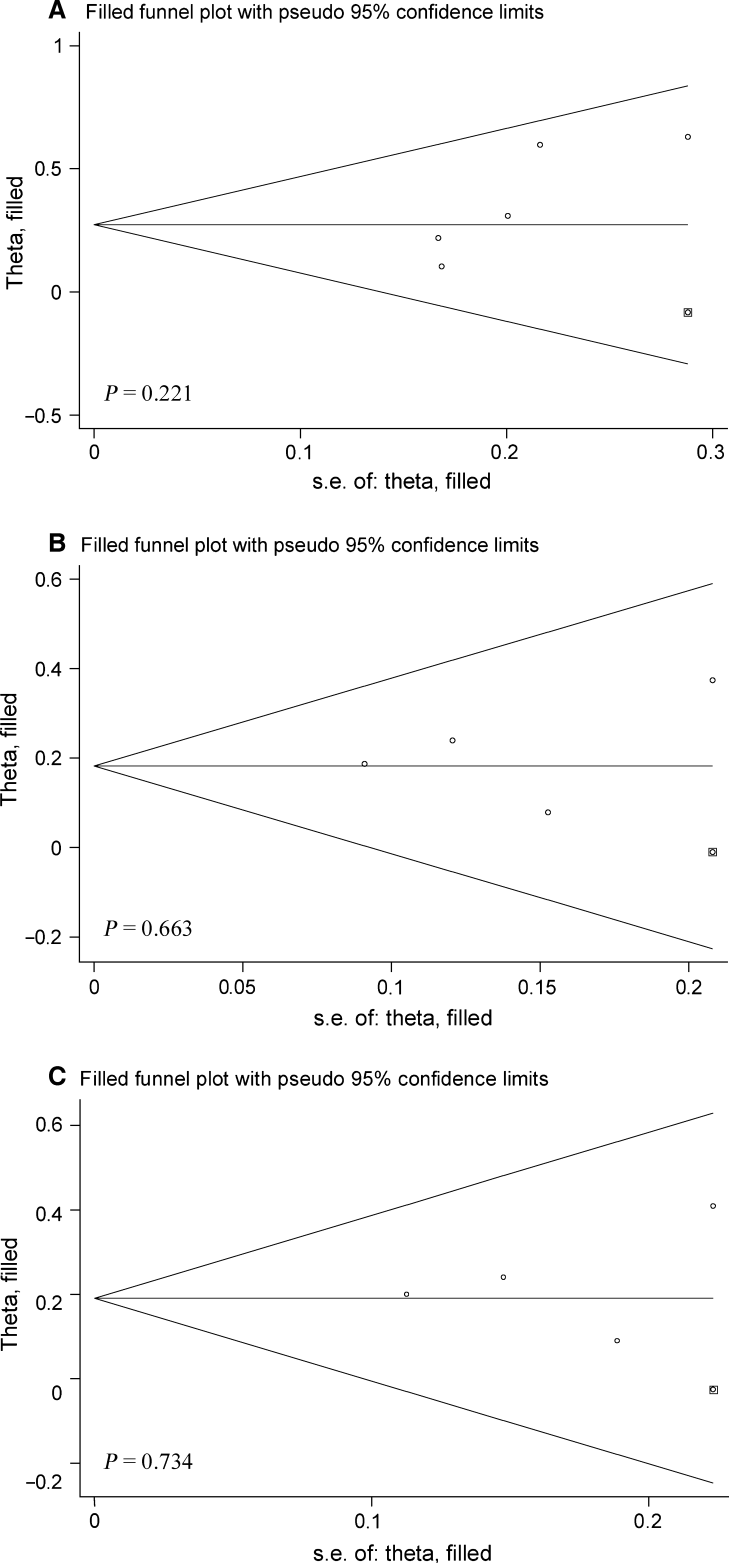
Filled funnel plots for Arg280His under allelic model (**A**) and Asp312Asn under allelic (**B**) and dominant (**C**) models.

Using the trim‐and‐fill method, one study was reported to be missing for Arg280His under allelic model and for Asp312Asn under both models (Fig. 1). After taking missing studies into account, the magnitude of association was weakened, and still there was statistical significance (adjusted OR, 95% CI, *P*: 1.32, 1.09–1.60, 0.004 for Arg280His under allelic model, and for Asp312Asn, 1.20, 1.07–1.35, 0.002 under allelic model and 1.21, 1.05–1.39, 0.008 under dominant model).

**Figure 2:**
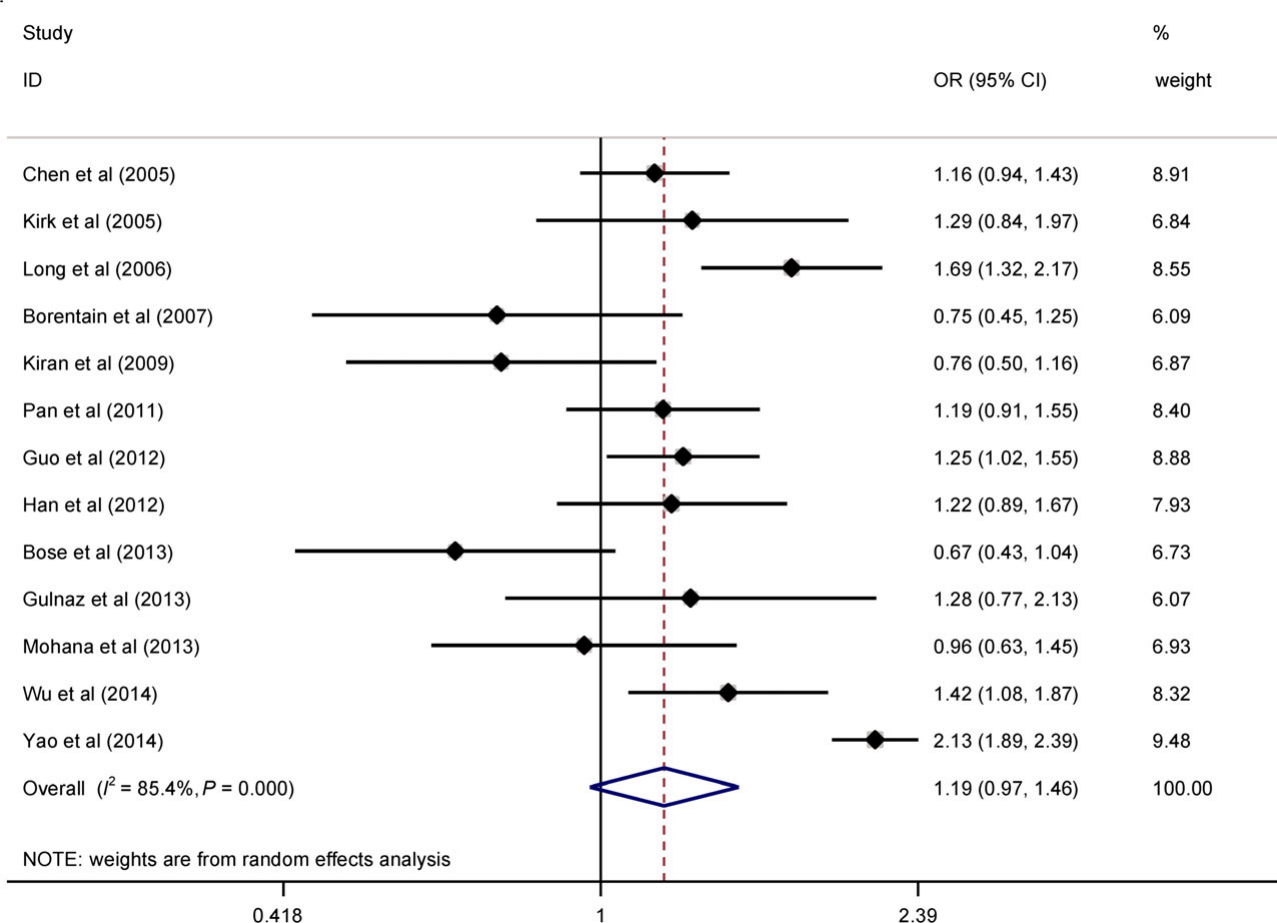
Forest plot of Arg399Gln variant in association with hepatocellular carcinoma risk.

In view of the limited number of qualified studies, stratified analyses were only undertaken for Arg399Gln (13 studies, Fig. 2) to seek possible sources of heterogeneity (Table 3). First, by country, the 399Gln allele was associated with the significant risk of hepatocellular carcinoma in Chinese subjects, especially from south China (OR, 95% CI, *P*: 1.57, 1.16–2.14, 0.004 under allelic model and 1.69, 1.18–2.42, 0.004 under dominant model), and no significance was found in subjects from the Indo‐Pakistani region and France. Second, after grouping studies by total sample size at a cut‐off of 500, there was no significance in smaller studies under both genetic models, but in larger studies, the 399Gln allele was associated with the significant risk of hepatocellular carcinoma under both allelic (OR, 95% CI, *P*: 1.48, 1.11–1.98, 0.007) and dominant (1.57, 1.12–2.19, 0.008) models. Third, regarding source of controls, significance was only identified for Arg399Gln in studies recruiting controls from populations (OR, 95% CI, *P*: 1.33, 1.06–1.68, 0.016 under allelic model and 1.45, 1.10–1.90, 0.008 under dominant model) rather than from hospitals. Fourth, upon stratification by genotyping method, Arg399Gln variant was not associated with hepatocellular carcinoma under both genetic models for studies using restriction fragment length polymorphism (RFLP) method and the others. Fifth, restricting analysis to studies with matched age between patients and controls identified significant association with hepatocellular carcinoma (OR, 95% CI, *P*: 1.28, 1.03–1.58, 0.026 under allelic model and 1.40, 1.10–1.78, 0.007 under dominant model). Sixth, to exclude the confounding impact of disproportionate hepatitis B virus infection, we analysed this association in studies with the comparable percentage of hepatitis B virus infection between the two groups, and there was enhanced association under both allelic (OR, 95% CI, *P*: 1.50, 1.08–2.10, 0.017) and dominant (1.70, 1.18–2.44, 0.004) models.

**Table 3:** Subgroup analyses of Arg399Gln variant in association with hepatocellular carcinoma

Subgroups	Models	Number of studies	OR	95% CI	*P*	*I* ^2^ (%)
Country						
North China	Allelic	3	1.23	1.06–1.42	0.006	0.0
	Dominant	3	1.34	1.08–1.65	0.007	0.0
South China	Allelic	4	1.57	1.16–2.14	0.004	89.3
	Dominant	4	1.69	1.18–2.42	0.004	87.9
Indo‐Pakistani	Allelic	4	0.87	0.67–1.13	0.304	27.2
	Dominant	4	0.80	0.59–1.10	0.167	0.0
France	Allelic	2	1.00	0.59–1.69	0.995	59.9
	Dominant	2	0.95	0.50–1.82	0.885	58.4
Source of controls						
Hospital	Allelic	5	0.95	0.74–1.24	0.726	37.9
	Dominant	5	0.94	0.72–1.22	0.621	0.0
Population	Allelic	8	1.33	1.06–1.68	0.016	87.6
	Dominant	8	1.45	1.10–1.90	0.008	84.4
Genotyping methods						
RFLP	Allelic	6	1.07	0.77–1.48	0.688	74.6
	Dominant	6	1.06	0.71–1.60	0.763	72.4
Others	Allelic	7	1.29	0.99–1.68	0.057	88.7
	Dominant	7	1.38	1.01–1.89	0.045	85.6
Matched status						
Matched	Allelic	4	1.28	1.03–1.58	0.026	86.2
	Dominant	4	1.40	1.10–1.78	0.007	81.2
Matched on HBV						
HBV‐matched	Allelic	4	1.50	1.08–2.10	0.017	91.1
	Dominant	4	1.70	1.18–2.44	0.004	87.4
Sample size						
<500	Allelic	8	1.03	0.85–1.25	0.733	52.2
	Dominant	8	1.05	0.82–1.34	0.685	38.0
≥500	Allelic	5	1.48	1.11–1.98	0.007	89.1
	Dominant	5	1.57	1.12–2.19	0.008	88.0

RFLP, restriction fragment length polymorphism; OR, odds ratio; 95% CI, 95% confidence interval.

Further in meta‐regression analysis, the percentages of hepatitis B virus infection in patients (*P* = 0.005) and controls (*P* = 0.008) were found to be significant confounders for the association between Arg399Gln variant and hepatocellular carcinoma risk.

## Discussion

The aim of this comprehensive meta‐analysis was to systematically assess the susceptible roles of six non‐synonymous coding variants in three DNA repair genes from the English literature. The key findings of this meta‐analysis are that *XPD* gene Asp312Asn was a candidate locus predisposing individuals to hepatocellular carcinoma, and for the widely evaluated variant Arg399Gln in *XRCC1* gene, country, sample size, hepatitis B virus infection and source of controls might confound its association with hepatocellular carcinoma.

It is widely accepted that hepatocellular carcinoma is a multifactorial disease, and generally, carcinogenesis can be promoted by a single dominant mutation leading to expression of an oncogene. It is of importance to capture targeted genetic culprits responsible for the functional changes of these oncogenes. The interrogation of non‐synonymous variants in coding region is a promising choice as they might change protein's function and consequently tailor susceptibility to disease 28. To increase the chance of identifying susceptibility loci, we focused on six non‐synonymous variants in three DNA repair genes, and 20 qualified studies systematically assessed their potential roles in susceptibility to hepatocellular carcinoma. Our overall analyses revealed that the mutation of Asp312Asn in *XPD* gene increased the risk of having hepatocellular carcinoma, irrespective of genetic models, and there was no indication of heterogeneity and publication bias. However, a note of caution should be sounded when interpreting this finding as only four studies were available for summarization, and moreover as we only analysed data in articles published in English language, the possible existence of publication bias cannot be excluded convincingly 29.

Although the overall association between *XRCC1* gene Arg399Gln and hepatocellular carcinoma was negative, further stratified analyses revealed that country, sample size, hepatitis B virus infection and source of controls constituted the potential sources of heterogeneity. It is of particular interest to notice that Arg399Gln variant was associated with the significant risk of hepatocellular carcinoma in Chinese subjects, especially from south China, suggesting the existence of genetic heterogeneity. This association was deemed robust as significance remained after restricting analysis to larger studies only. Moreover, it is widely recognized that the coinfection of hepatitis B virus is an established risk factor for hepatocellular carcinoma, and this infection was unlikely able to cloud the susceptibility of Arg399Gln variant to hepatocellular carcinoma. On the basis of these observations, our meta‐analytical findings suggested that *XRCC1* gene Arg399Gln was another susceptibility locus for hepatocellular carcinoma in Chinese. However, considering the limited number of qualified studies, this result cannot be directly extrapolated to the other ethnic groups, and further investigations in groups of other continents are timely required.

Several possible limitations should be acknowledged in this meta‐analysis. First, only English articles were retrieved, and selection bias cannot be totally ruled out, although our trim‐and‐fill method revealed a low probability of publication bias. Second, only six non‐synonymous variants from three DNA repair genes were meta‐analysed, and other functional variants such as in the promoter regions of other relevant genes also deserved attention pending sufficient published data. Third, besides Arg399Gln, there were limited numbers of qualified studies for the other examined variants, which precluded further exploration on heterogeneity by using stratified and meta‐regression analyses. Fourth, all involved studies were retrospective in nature, and it is intriguing to see whether the two significant variants were associated with the relapse, metastasis and survival in patients with hepatocellular carcinoma following hepatectomy.

In conclusion, we through a comprehensive meta‐analysis demonstrated that *XPD* gene Asp312Asn and *XRCC1* gene Arg399Gln might be candidate susceptibility loci for hepatocellular carcinoma. Considering the ubiquity of genetic heterogeneity and in view of small sample sizes involved, our findings should be considered to be preliminary until being replicated or confirmed in other larger, well‐designed studies in the future. For practical reasons, successful confirmation of our findings would facilitate the identification of individuals at high risk for developing hepatocellular carcinoma in future clinical screening.

## Supporting information


**Figure S1** The selection process of qualified articles in this meta‐analysis.

**Table S1** The PRISMA checklist.
