# Supplementary figures and images for: The association of six non‐synonymous variants in three DNA repair genes with hepatocellular carcinoma risk: a meta‐analysis
Source: J Cell Mol Med. 2016 Jun 16;20(11):2056–63. doi: 10.1111/jcmm.12896 (PMC5082408; doi:10.1111/jcmm.12896)

**Supplementary Figure S1.** The selection process of qualified articles in this meta-analysis

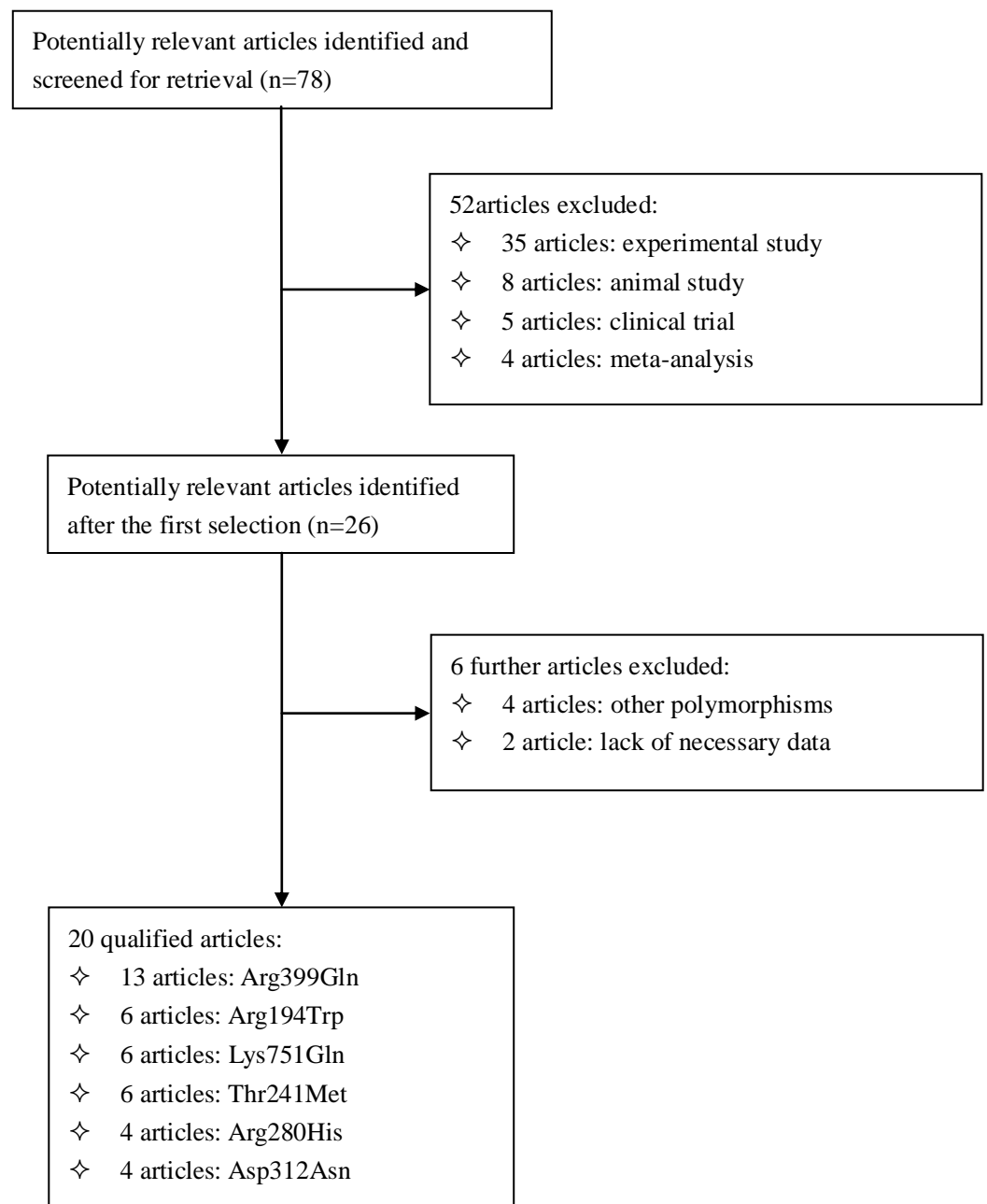

Supplement: Supplementary file 1 — Figure S1 The selection process of qualified articles in this meta‐analysis. [file JCMM-20-2056-s001.pdf]
